# Outcome and complication comparison for intramedullary nail versus open reduction internal fixation in humeral diaphyseal fractures for 2800 matched patient pairs utilizing the Nationwide Readmissions Database

**DOI:** 10.1186/s13018-023-03663-2

**Published:** 2023-06-20

**Authors:** Kirsi S. Oldenburg, Megan E. Welsh, Jonathan Brett Goodloe, Richard J. Friedman, Josef K. Eichinger

**Affiliations:** Department of Orthopaedics, Medical University of South Carolina, 96 Jonathan Lucas Street, CSB 708, Charleston, SC 29425 USA

**Keywords:** Intramedullary nail fixation (IMN), Open reduction internal fixation (ORIF), Nationwide Readmissions Database (NRD), Revision, Outcomes, Humeral shaft fracture

## Abstract

**Introduction:**

Open reduction internal fixation (ORIF) and intramedullary nail fixation (IMN) are the predominant repair methods for operative treatment of humeral diaphyseal fractures; however, the optimal method is not fully elucidated. The purpose of this study was to analyze whether IMN or ORIF humeral diaphyseal surgeries result in a significantly higher prevalence of adverse outcomes and whether these outcomes were age dependent. We hypothesize there is no difference in reoperation rates and complications between IMN and ORIF for humeral diaphyseal fractures.

**Methods:**

Data collected from 2015 to 2017 from the Nationwide Readmissions Database were evaluated to compare the prevalence of six adverse outcomes: radial nerve palsy, infections, nonunion, malunion, delayed healing, and revisions. Patients treated for a primary humeral diaphyseal fracture with either IMN or ORIF were matched and compared (*n* = 2,804 pairs). Patients with metastatic cancer were excluded.

**Results:**

Following an ORIF procedure, there was a greater odds of undergoing revision surgery (*p* = 0.03) or developing at least one of the complications of interest (*p* = 0.03). In the age-stratified analysis, no significant differences were identified in the prevalence of adverse outcomes between the IMN and ORIF cohorts in the 0–19, 20–39, and 40–59 age groups. Patients who were 60 + had 1.89 times the odds of experiencing at least one complication and 2.04 times the odds of undergoing a revision after an ORIF procedure versus an IMN procedure (*p* = 0.03 for both).

**Discussion:**

IMN and ORIF for humeral diaphyseal fractures are comparable in regard to complications revision rates in patients under the age of 60. Meanwhile, patients 60 + years show a statistically significant increase in the odds of undergoing revision surgery or experiencing complications following an ORIF. Since IMN appears to be more beneficial to older patients, being 60 + years old should be considered when determining fracture repair techniques for patients presenting with primary humeral diaphyseal fractures.

**Level of Evidence** III.

**Supplementary Information:**

The online version contains supplementary material available at 10.1186/s13018-023-03663-2.

## Introduction

Due to the aging US population, the prevalence of humeral diaphyseal fractures is predicted to increase significantly between now and 2030 [1]. In 2008, humeral diaphyseal fractures were the least common humeral fracture presenting to US emergency departments [1]. Humeral diaphyseal fractures in the elderly population are typically caused by a simple fall [2, 3], while in younger patients such fractures are usually caused by high-energy trauma [4]. A majority of the humeral diaphyseal fractures experienced in younger patients occur in males, while females compose a greater proportion of fractures that occur in the older generations [3, 5, 6].

Humeral diaphyseal fractures can be treated both surgically and non-surgically. Non-surgical treatment involves either a sling, cast, or a functional brace. Surgical interventions include intramedullary nail (IMN) fixation, open reduction internal fixation (ORIF), and minimally invasive percutaneous osteosynthesis (MIPO) [7]. IMN fixation allows for preservation of blood supply to the fracture site with the goal of relative stability and secondary bone healing. Open reduction internal fixation often utilizes a combination of plate and screw fixation with interfragmentary fixation with the goal of achieving primary bone healing through compression.

While the majority of humeral diaphyseal fractures can be treated non-operatively with high rates of healing [8], operative repair is considered for a variety of indications. These include open fractures, multiple extremity injuries, associated neurovascular injury, failure of non-operative treatment, pain, and unacceptable fracture displacement. ORIF and IMN are the two predominant forms of operative repair, but the optimal method and the associated complications are not fully elucidated. According to an analysis of the American Board of Orthopaedic Surgery Part II operative database by Gottschalk et al., between 2004 and 2013 ORIF became a more popular technique for treating humeral diaphyseal fractures compared to IMN [9]. However, the literature is inconclusive as to which surgical method is superior to the other. Some studies report that ORIF is favored over IMN [10–12], while others indicate that there is no difference in results between the two methods [13, 14]. One meta-analysis analyzed randomized clinical trials and observational studies to compare outcomes of the two procedures. This analysis concluded that IMN demonstrated a lower risk of infection, postoperative radial nerve palsy, shorter operation time and shorter time to union compared to ORIF. However, IMN had higher re-intervention rates due to shoulder impingement after surgery. Overall, the differences between IMN and ORIF were small and optimal results were achieved with both procedures [15]. Understanding which surgical treatment provides the best functional and radiological outcomes for patients is necessary in order to ensure the best care for patients postoperatively. If there truly is no significant difference in patient outcomes between IMN and ORIF, other factors such as differences in cost and operation time should be considered.

Due to the aforementioned disagreement in the literature as to which surgical treatment for humeral diaphyseal fractures is most beneficial to patients, we sought to determine differences in complication rates by querying the Nationwide Readmissions Database (NRD). Additionally, our study sought to provide valuable insight into the possible age dependency of adverse outcomes following an IMN or ORIF humeral diaphyseal procedure by further stratifying our results by different age cohorts. The purpose of this study is to determine if there are any significant differences in outcomes between IMN and ORIF for humeral diaphyseal fractures. We hypothesize there is no difference in regards to reoperation rates and complications between IMN and ORIF for humeral diaphyseal fractures.

## Methods

### Database

Patients were extracted from the Healthcare Cost and Utilization Project Nationwide Readmission Database (HCUP NRD). The HCUP NRD is a national deidentified database that contains clinical and nonclinical data from inpatient visits occurring in twenty-eight different states. HCUP NRD in its unweighted form accounts for 58.2% of hospitalizations in the USA. Data from years 2015–2017 were used for this study, and no IRB approval was needed because the research involves the collection or study of publicly existing data with de-identified subjects.

### Sample selection

Patients were selected from the HCUP NRD based on if they received an IMN or ORIF procedure for a primary humeral diaphyseal fracture (Additional file 1: Table S1). In total, there were 31,038 unique cases. In an attempt to only select cases representing a "primary" visit for an IMN or ORIF procedure, all cases that also contained an outcome of interest within the same hospital visit of the procedure were removed. The outcomes of interest for this study included injury of the radial nerve at the upper arm level, infection and inflammatory reaction of the humerus due to an internal fixation device, nonunion of a humeral diaphyseal fracture, malunion of a humeral diaphyseal fracture, delayed healing of a humeral diaphyseal fracture, and revision of a previous IMN or ORIF procedure. Patients who had bilateral IMN or ORIF procedures were removed. Additional exclusion factors included: undergoing both IMN and ORIF procedure in the same hospitalization, a sequela designation for their humeral diaphyseal fracture, death during the original visit, a missing death status, and/or a metastatic cancer diagnosis. In total, there were 3,093 original IMN visits and 24,337 original ORIF visits available for analysis. All follow-up visits pertaining to these patients within the calendar year of their original IMN or ORIF visit were pulled.

### Revisions

A revision occurred if: (1) there was an ICD-10 code for a revision, (2) the patient had their IMN or ORIF ware removed in addition to another IMN or ORIF procedure, (3) or if the patient received an additional IMN or ORIF procedure on the same arm as the original procedure.

### Control matching

IMN and ORIF patients were matched based on the following predictors: sex, age, primary expected payer, median household income, and whether they were a resident in the state of the procedure. The sampling occurred without replacement, with randomized case order when drawing matches, and gave priority to exact matches. The match tolerance was set at 0 for all variables. Case–control matching was run with IBM SPSS Statistics for Windows, version 25 (IBM Corp., Armonk, N.Y., USA). The matching process did not include any patients missing a value in the five aforementioned predictors. In total, 2,804 ORIF and IMN cases, and their corresponding readmissions, were available for analysis.

### Statistical analysis

All analyses were conducted in RStudio version 1.2.5033 (RStudio, Inc., Boston, MA, USA. URL http://www.rstudio.com/). Demographics, comorbidities, and unadjusted differences in outcomes were analyzed using the McNemar’s test with the continuity correction. The variables analyzed with the McNemar’s test all had one degree of freedom, except for the quarter of hospitalization which had 6 degrees of freedom. Further analysis involved stratifying the patients by age, with the following age groups: 0–19, 20–39, 40–59, and 60 + . Analyses controlling for confounding comorbidities were run using a conditional logistic regression through the clogistic function of the Epi package. Anemia deficiency, lymphoma, and peripheral vascular disorders were the potentially confounding comorbidities identified because they were significantly different between patients who underwent an IMN or ORIF procedure. Individual analysis of the comorbidities occurred for the age stratified groups as well. Adjusted analyses could not be run for any outcomes where at least one group had zero patients with the outcome. Additionally, statistical analyses were not run for the radial nerve palsy outcome as both proportions were zero. Results were statistically significant if the *p*-value was less than 0.05.

## Results

The average age of the two cohorts was 59.3 years and primarily female (60.2%) (Table 1). A majority were insured by Medicare (53.7%), and almost a third were in a zip code where the median household income was in the 0-25th percentile (lowest income) (Table 1). Tobacco use and discharge quarter were, respectively, not significantly different between the IMN and ORIF matched cohorts (Table 1).

**Table 1:** Demographic data for matched pairs composed of patients who underwent an IMN or ORIF procedure

Demographic factors	IMN (*n* = 2,804)	ORIF (*n* = 2,804)	Matched pair has outcome^#^ (*n* = 2,804 pairs)	*p*-value
Age (average years ± standard deviation)	59.30 ± *23.5*	59.30 ± *23.5*	–	–
Females	1,687 (60.2%)	1,687 (60.2%)	–	–
Home state is the same as hospital state	2,679 (95.5%)	2,679 (95.5%)	–	–
*Primary expected payer*				
Medicare	1505 (53.7%)	1505 (53.7%)	–	–
Medicaid	371 (13.2%)	371 (13.2%)	–	–
Private insurance	697 (24.9%)	697 (24.9%)	–	–
Self-pay	94 (3.4%)	94 (3.4%)	–	–
No charge	5 (0.2%)	5 (0.2%)	–	–
Other	132 (4.7%)	132 (4.7%)	–	–
*Median household income of patient’s zipcode*				
0–25^th^ percentile	843 (30.1%)	843 (30.1%)	–	–
26–50^th^ percentile	777 (27.7%)	777 (27.7%)	–	–
51–75^th^ percentile	667 (23.8%)	667 (23.8%)	–	–
76–100^th^ percentile	517 (18.4%)	517 (18.4%)	–	–
Tobacco use^*^	441 (15.7%)	438 (15.6%)	90 (3.2%)	0.9
*Discharge quarter*				
January–March	609 (21.7%)	610 (21.8%)	739 (26.4%)	0.4
April–June	639 (22.8%	605 (21.6%)		
July–September	621 (22.1%)	620 (22.1%)		
October–December	935 (33.3%)	969 (34.6%)		

*Defined by an ICD-10 diagnosis code of E66.0, E66.01, E66.09, E66.1, E66.2, E66.3, E66.8, or E66.9

^#^ Matched pair refers to the pair created by matching demographic factors [age, sex, their primary expected payer, median household income, and patient is a resident where hospitalized] of patients who underwent a IMN or ORIF procedure

Both patients within the pair must have the demographic factor to be reported in this column

*Defined by an ICD-10 diagnosis code of: Z72.0, Z87.891, and/or V15.82

The IMN cohort had a significantly greater number of patients with deficiency anemia (*p* < 0.001), lymphoma (*p* < 0.001), and peripheral vascular disorders (*p* = 0.04) (Table 2).

**Table 2:** Preoperative comorbidities for patients who underwent an IMN or ORIF procedure for a diaphyseal fracture

Comorbidities	IMN (*n* = 2,804)	ORIF (*n* = 2,804)	Matched pair has outcome^#^ (*n* = 2,804 pairs)	*p*-value
AIDS	5 (0.2%)	5 (0.2%)	0 (0%)	1
Alcohol abuse	235 (8.4%)	216 (0.2%)	31 (1.1%)	0.4
Chronic pulmonary disease	454 (16.2%)	452 (16.1%)	84 (3.0%)	1
Diabetes, uncomplicated	396 (14.1%)	367 (13.1%)	64 (2.3%)	0.3
Liver disease	83 (3.0%)	95 (3.4%)	7 (0.25%)	0.4
Rheumatoid arthritis/collagen vascular diseases	78 (2.8%)	91 (3.2%)	3 (0.11%)	0.3
Hypertension (complicated and uncomplicated)	1,502 (53.6%)	1,482 (52.9%)	1,015 (36.2%)	0.5
Metastatic cancer	0 (0%)	0 (0%)	0 (0%)	–
Valvular disease	129 (4.6%)	138 (4.9%)	8 (0.29%)	0.6
Diabetes, chronic complications	262 (9.3%)	267 (9.5%)	32 (1.1%)	0.9
Deficiency anemia	540 (19.3%)	404 (14.4%)	101 (3.6%)	** < 0.001**
Chronic blood loss anemia	35 (1.2%)	38 (1.4%)	0 (0%)	0.8
Congestive heart failure	202 (7.2%)	209 (7.5%)	33 (1.2%)	0.7
Coagulopathy	140 (5.0%)	145 (5.2%)	12 (0.43%)	0.8
Depression	396 (14.1%)	410 (14.6%)	72 (2.6%)	0.6
Hypothyroidism	386 (13.8%)	369 (13.2%)	81 (2.9%)	0.5
Fluid and electrolytes disorders	583 (20.8%)	561 (20.0%)	139 (5.0%)	0.5
Pulmonary circulation disorders	17 (0.6%)	15 (0.5%)	0 (0%)	0.9
Renal failure	267 (9.5%)	247 (8.8%)	42 (1.5%)	0.4
Peptic ulcer disease, excluding bleeding	16 (0.6%)	14 (0.5%)	1 (0.04%)	0.9
Drug abuse	72 (2.6%)	86 (3.1%)	5 (0.18%)	0.3
Lymphoma	197 (7.0%)	37 (1.3%)	3 (0.11%)	** < 0.001**
Neurological disorders	279 (10.0%)	287 (10.2%)	32 (1.1%)	0.8
Peripheral vascular disorders	117 (4.2%)	87 (3.1%)	3 (0.1%)	**0.04**
Obesity	413 (14.7%)	377 (13.4%)	68 (2.4%)	0.2
Patients with paralysis	63 (2.2%)	65 (2.3%)	3 (0.1%)	0.9

^#^ Matched pair refers to the pair created by matching demographic factors [age, sex, their primary expected payer, median household income, and patient is a resident where hospitalized] of patients who underwent a IMN or ORIF procedure

Both patients within the pair must have the demographic factor to be reported in this column

Bolded values are significant

In the unadjusted comparison of outcomes between the IMN and ORIF pairs, there were no statistically significant differences, although the greater prevalence of revisions in the ORIF cohort was borderline significant (*p* = 0.05) (Table 3).

**Table 3:** Comparing the proportion of various outcomes between the IMN and ORIF patients

Outcome^+^	IMN (*n* = 2,804)		ORIF (*n* = 2,804)		*p*-value
	*n*	Complication Rate^#^	*n*	Complication rate^#^	
Radial nerve palsy	0 (0%)	0	0 (0%)	0	−
Infection	5 (0.2%)	17.8	14 (0.5%)	49.9	0.07
Nonunion	12 (0.4%)	42.8	9 (0.3%)	32.1	0.7
Malunion	2 (0.1%)	7.13	0 (0%)	0	0.5
Delayed healing	6 (0.2%)	21.4	5 (0.2%)	17.8	1
Revision	33 (1.2%)	117.7	52 (1.9%)	185.4	0.05
*Any complication	44 (1.6%)	156.9	65 (2.3%)	231.8	0.06

+ There were zero pairs where both patients within the pair had the outcome of interest

^*^Any complication was defined by if the patient had either radial nerve palsy, an infection, a nonunion, a malunion, delayed healing, and/or a revision

− Because the proportion of patients with radial nerve palsy was zero in both groups, no statistics were run

^#^ per 10,000 people

After controlling for the effects of anemia deficiency, lymphoma, and peripheral vascular disorders, there was no significant differences between the IMN and ORIF procedures for infection or nonunion. However, there were significantly greater odds of receiving a revision or experiencing any complication after undergoing an ORIF procedure versus an IMN procedure (*p* = 0.03 for both) (Table 4).

**Table 4:** Potential confounding comorbidities when evaluating the outcomes of patients who underwent an IMN or ORIF

Outcomes^~^	*β*	Standard error (*β*)	Odds ratio	*p*-value
Radial nerve palsy	–	–	–	–
Infection	0.81	0.60	2.25	0.18
Nonunion	− 0.13	0.52	0.88	0.80
Malunion^**^	–	–	–	–
Delayed healing	− 0.13	0.62	0.88	0.84
Revision	0.51	0.24	1.66	**0.03**
*Any complication	0.46	0.21	1.58	**0.03**

Bolded *p*-values show statistically significant results

*Any complication was defined by if the patient had either radial nerve palsy, an infection, a nonunion, a malunion, delayed healing, and/or a revision

**Iteration limit was exceeded, analysis could not be run

~ Adjusted results controlling for anemia deficiency, lymphoma, and peripheral vascular disorders

For the age-stratified analysis, no comorbidities were significantly different between the IMN and ORIF patients in the 0–19 cohort nor the 20–39 cohort. IMN patients in the 40–59 age group demonstrated a significantly greater prevalence of deficiency anemia and lymphoma (*p* < 0.001 for both), and a borderline significantly greater prevalence of obesity (*p* = 0.05) (Table 5). IMN patients in the 60 + age group demonstrated significantly greater prevalence of deficiency anemia and lymphoma (*p* < 0.001 for both), and a borderline significantly greater prevalence of peripheral vascular disorders (*p* = 0.05) (Table 5).

**Table 5:** Comorbidities that were significantly different between patients in the age-stratified analysis

Comorbidities	IMN	ORIF	Matched Pair Has Outcome^#^	*p*-Value
*Ages 0–19 (n* = *239 per group)*				
**–**	–	–	–	–
*Ages 20–39 (n* = *344 per group)*				
**–**	–	–	–	–
*Ages 40–59 (n* = *523 per group)*				
Deficiency anemia	90 (17.2%)	48 (9.2%)	6 (1.1%)	** < 0.001**
Lymphoma	41 (7.8%)	7 (1.3%)	0 (0%)	** < 0.001**
Obesity	107 (20.4%)	82 (15.6%)	19 (3.6%)	0.05
*Ages 60* + *(n* = *1697 per group)*				
Deficiency anemia	421 24.8%)	330 (19.4%)	94 (5.5%)	** < 0.001**
Lymphoma	154 (9.1%)	30 (1.8%)	3 (0.2%)	** < 0.001**
Peripheral vascular disorders	93 (5.5%)	67 (3.9%)	2 (0.1%)	0.05

^#^Matched pair refers to the pair created by matching demographic factors [age, sex, their primary expected payer, median household income, and patient is a resident where hospitalized] of patients who underwent a IMN or ORIF procedure

Both patients within the pair must have the demographic factor to be reported in this column

Bolded *p*-values are significant

When comparing the prevalence of adverse outcomes in the unadjusted age-stratified analysis, there were no significant differences in adverse outcomes between the IMN and ORIF cohorts in the 0–19, 20–39, and 40–59 age groups (Table 6). In the 60 + age group, there was a significantly greater prevalence of revisions and increased risk of experiencing any type of complication in the ORIF cohort (*p* = 0.04 for both) (Table 6).

**Table 6:** Age stratified unadjusted differences in outcomes between patients who received an IMN or ORIF

Outcome	IMN		ORIF		*p*-Value
	*n* (%)	Complication Rate^#^	*n* (%)	Complication Rate^#^	
*Ages 0–19 (n* = *239 per group)*					
Radial nerve palsy	0 (0%)	0	0 (0%)	0	–
Infection	2 (0.8%)	83.7	0 (0%)	0	0.5
Nonunion	1 (0.4%)	41.8	0 (0%)	0	1
Malunion	0 (0%)	0	0 (0%)	0	–
Delayed healing	0 (0%)	0	0 (0%)	0	–
Revision	0 (0%)	0	2 (0.8%)	83.7	0.5
*Any complication	3 (1.3%)	125.5	2 (0.8%)	83.7	1
*Ages 20–39 (n* = *344 per group)*					
Radial nerve palsy	0 (0%)	0	0 (0%)	0	–
Infection	1 (0.3%)	29.1	1 (0.3%)	29.1	1
Nonunion	2 (0.6%)	58.1	0 (0%)	0	0.5
Malunion	0 (0%)	0	0 (0%)	0	–
Delayed healing	1 (0.3%)	29.1	0 (0%)	0	1
Revision	2 (0.6%)	58.1	4 (1.2%)	116.2	0.7
*Any complication	3 (0.9%)	87.2	5 (1.5%)	145.3	0.7
*Ages 40–59 (n* = *523 per group)*					
Radial nerve palsy	0 (0%)	0	0 (0%)	0	–
Infection	0 (0%)	0	5 (1.0%)	95.6	0.07
Nonunion	5 (1.0%)	95.6	1 (0.2%)	19.1	0.2
Malunion	0 (0%)	0	0 (0%)	0	–
Delayed healing	2 (0.4%)	38.2	3 (0.6%)	57.4	1
Revision	13 (2.5%)	248.6	12 (2.3%)	229.4	1
*Any complication	15 (2.9%)	286.8	18 (3.4%)	344.2	0.7
*Ages 60* + *(n* = *1697 per group)*					
Radial nerve palsy	0 (0%)	0	0 (0%)	0	–
Infection	2 (0.1%)	11.8	8 (0.5%)	47.1	0.1
Nonunion	4 (0.2%)	23.6	8 (0.5%)	47.1	0.4
Malunion	2 (0.1%)	11.8	0 (0%)	0	0.5
Delayed healing	3 (0.2%)	17.7	2 (0.1%)	11.8	1
Revision	18 (1.1%)	106.1	34 (2.0%)	200.4	**0.04**
*Any complication	23 (1.4%)	135.5	40 (2.4%)	235.7	**0.04**

Bolded *p*-values show statistically significant results

*Any complication was defined by if the patient had either radial nerve palsy, an infection, a nonunion, a malunion, delayed healing, and/or a revision

^#^ Per 10,000 people

In the age-stratified adjusted analysis, there again were no significant differences in adverse outcomes between the IMN and ORIF cohorts in the 0–19, 20–39, and 40–59 age groups (Table 7). Similarly, in the 60 + age group, there were no significant differences in the prevalence of infections, nonunions, malunions, or delayed healing between the two procedures. However, in the 60 + age group, patients who underwent an ORIF procedure had 2.04 times the odds of undergoing a revision and 1.89 times the odds of experiencing any complication compared to their IMN matched cohort (*p* = 0.03 for both) (Table 7).

**Table 7:** Age adjusted odds ratios in outcomes between patients who received an IMN or ORIF for a humeral diaphyseal fracture

Outcomes^~^	*β*	Standard error (*β*)	Odds ratio	*p*-value
*Ages 0–19*				
*Any complication	− 0.41	0.91	0.67	0.66
*Ages 20–39*				
Infection	0.00	1.41	1.00	1.00
Revision	0.69	0.87	2.00	0.42
*Any complication	0.51	0.73	1.67	0.48
*Ages 40–59*				
Nonunion	− 1.10	1.15	0.33	0.34
Delayed healing	< 0.01	1.41	1.00	1.00
Revision	< 0.01	0.43	1.00	1.00
*Any complication	0.22	0.39	1.24	0.57
*Ages 60* +				
Infection	0.92	0.84	2.50	0.27
Nonunion	1.10	0.82	3.00	0.18
Delayed healing	− 1.10	1.15	0.33	0.34
Revision	0.71	0.33	2.04	**0.03**
*Any complication	0.64	0.29	1.89	**0.03**

Bolded *p*-values show statistically significant results

*Any complication was defined by if the patient had either radial nerve palsy, an infection, a nonunion, a malunion, delayed healing, and/or a revision

~ Analysis of ages 0–19 and 20–39 had no predictor variables outside of being a Nail or ORIF case; ages 40–59 were adjusted for deficiency anemia, lymphoma, and obesity status; ages 60 + were adjusted for deficiency anemia, lymphoma, and peripheral vascular disorders

## Discussion

This study utilized the NRD to compare revision and complication rates among patients treated with ORIF and IMN of humeral diaphyseal fractures. Overall, the two fracture fixation methods were fairly comparable. However, after controlling for the effects of anemia deficiency, lymphoma, and peripheral vascular disorders, there were significantly greater odds of requiring revision surgery or experiencing any complication with ORIF versus IMN (*p* = 0.03 for both). When stratified by age, patients over the age of 60 who underwent ORIF had 2.04 times higher odds of undergoing revision surgery and 1.89 times higher odds of experiencing any complication when compared to the IMN cohort. Since the statistical significance is affected by the large sample size it is difficult to explain the findings as clinically significant in terms of preferred treatment, however the increased risk associated with ORIF in older patients should still be considered when operating on the older population. The finding highlights why bone density is a key argument in the controversy regarding optimal PHF treatment in the geriatric population. Regardless of age, if a person has sufficient bone quality, then outcomes should be the same, but a characteristic prominent in the older population is a decrease in bone density over time [16]. The increased odds of a revision or complication in the 60 plus age group is important because it emphasizes the need for good bone quality in an ORIF procedure. Based on the results of these findings, older patients with diminished quality of bone may benefit from the use of a nail rather than plating for diaphyseal fractures. Technical advances in the use of the IMN nail are popularizing this technique as an easier, more reliable, less invasive surgery, with a load sharing biomechanical advantage [17[. Surgeons should also recognize the benefits of IMN as a less invasive procedure with potentially less blood loss particularly when operating on older patients. While current literature provides conflicting information on which procedure requires the longest amount of time, there is less blood loss associated with IMN than ORIF. IMN requires a smaller incision than what is required for plating, with less associated blood loss [18, 19]. These data add to the growing body of literature comparing the two surgical methods in an attempt to optimize outcomes for humeral diaphyseal fractures.

Over the past decade, meta-analyses sought to better delineate outcomes from IMN versus ORIF of humeral diaphyseal fractures, with great discordance among their findings. In 2013, Ma et al. and Liu et al. reported that both IMN and ORIF demonstrated no statistical difference between the prevalence of radial nerve injuries, infections, and fracture union [20, 21]. The results of our study are consistent with those of Ma et al. and Liu et al. However, Liu et al. reported that IMN patients experienced a higher rate of delayed healing, which was not found in the present study [21]. In 2015, Zhao et al. performed a systematic review of the overlapping meta-analyses and concluded that ORIF is superior to IMN largely based on the decreased risk of shoulder impingement, despite their findings demonstrating no difference in fracture union rates, radial nerve injury, and infection rates [22]. Unfortunately, many studies in the literature are limited by small samples sizes and vary in whether or not consideration was given to how comorbidities may affect the results. Kurup et al. acknowledged the low quality of evidence that was found in both their own systematic review and systematic reviews published by other authors [23].

McCormack et al. published a small prospective randomized control trial comparing IMN to ORIF and found no significant difference in ASES scores, VAS scores, strength, range of motion nor return to activity, but noted that the IMN cohort experienced a higher rate of complications and secondary procedures [24]. In 2011, a Cochrane database systematic review analyzed the outcomes of 260 humeral diaphyseal fractures treated with either IMN and ORIF and found no significant difference in fracture union rates, however, a significantly greater incidence of shoulder impingement and removal of instrumentation occurred with IMN [23]. The American Board of Orthopaedic Surgery surveyed surgeons sitting for part two of their boards and found a shift in utilization of IMN to ORIF for humeral diaphyseal fractures, with 42.9% IMN fixation in 2004 to 21.2% IMN fixation in 2013 [9]. Despite the trending decreased use of IMN, Gottschalk et al. reported that IMN treatment resulted in lower complications rates for infection and radial nerve palsy, with no significant difference in union rates, when compared to ORIF [9]. In contrast to previous studies, the current study found higher rates of revision surgery and complications in patients 60 years or older who underwent ORIF for humeral diaphyseal fractures.

One other study utilized the NRD to analyze IMN and ORIF humeral diaphyseal procedures. Merrill et al. evaluated length of stay and 30-day readmission rates after humeral diaphyseal fractures treated with IMN versus ORIF and found that 30-day readmission or length of stay was not affected by the procedure type, yet was impacted by comorbid conditions apart from the surgery [13]. To our knowledge, no prior study has used the NRD to compare specific adverse outcomes following IMN or ORIF humeral diaphyseal procedures, such as nonunion or infections, nor has any study analyzed outcome data within specific age groups.

The present study also controls for comorbidities, such as peripheral vascular disorders, that may potentially compromise or delay healing. Conducting analyses within four different age groups and controlling for comorbidities may account for and mitigate the discordance previously shown in the literature. According to our results, patients without metastatic cancer who are between the ages of 0–59 can undergo either. Nailing or plating of a diaphyseal humeral fracture can expect equivalent short-term results and complication risks. For the 60 + age group, physicians may consider the implications of their patient being at an increased risk for experiencing adverse outcomes after ORIF procedures on the humeral diaphyseal. The primary factor for the increased risk is likely bone quality as a function of age-related development of osteoporosis. While the results of the study do not evaluate the type of plate material, the length of plate, or the use of locking screws, plating in general has a higher rate of failure. If in fact osteoporosis is the primary factor resulting in greater failure or complication rates, then either an intramedullary implant or a plate screw construct that increases the working length of the construct should be considered. Overall, surgeons should consider age as a factor when determining which procedure to utilize on their patients presenting with a primary humeral diaphyseal fracture for patients over the age of 60 or when there is a concern regarding the quality of a patient’s bone.

Our study, and the NRD, are not without limitations. The NRD does not contain information pertaining to pre-operative assessments, which may guide decision making regarding the type of fixation used. The surgeon’s expertise in each procedure could not be evaluated, and this study did not specify the specific fracture type beyond diaphyseal fracture; however, these limitations were likely negated by the large sample size. Also, there is no information regarding the type of IMN, nailing method (antegrade vs. retrograde) or ORIF fixation device used for fixation. Specifically, it does not address the technique of plating with regards to length of construct, type and size of plate, type and number of screws (locking, non-locking), or method of fracture compression (standard AO technique versus bridge plating versus utilization of an articulated tensioning device). Additionally, patients treated on an outpatient basis were not included in the NRD, creating a potential selection bias. The NRD is also limited in terms of varying follow-up time postoperatively based on when a surgery occurred in relationship to the calendar year. Unfortunately, all cases presenting with radial nerve palsy at the original visit of interest were removed due to the inability to delineate whether the radial nerve palsy was caused by the procedure or the fracture itself. The effect of IMN on shoulder function and the effect of ORIF on triceps and elbow function are not elucidated with our analysis, nor within the NRD. Finally, the McNemar's test is known to be overly conservative, possibly underestimating the significance of the results [25].

## Conclusion

This study enhances the available literature comparing humeral diaphyseal fracture treatment options with IMN versus ORIF by addressing both the odds of developing postoperative adverse outcomes and the age dependency of experiencing specific adverse outcomes. Overall, our hypothesis of no statistically significant differences between IMN and ORIF was partially correct, with patients’ age largely influencing the results. Both methods of fixation appear to be effective for treating primary humeral diaphyseal fractures in patients without metastatic disease between the ages of 0–59, while patients 60 years or older experience a statistically significant increase in the odds of undergoing revision surgery or experiencing a complication following ORIF versus those receiving IMN. Thoughtful decision making and informed discussions regarding the risks and benefits of each method should be conducted with patients prior to surgical fixation.

## Supplementary Information

**Additional file 1. Table 1.** ICD-10 Codes for Identifying IMN and ORIF Cases.

## Data Availability

The datasets generated and analyzed during this study are available in the Nationwide Readmissions Database: https://www.hcup-us.ahrq.gov/nrdoverview.jsp.
