# Supplementary material for: Outcome and complication comparison for intramedullary nail versus open reduction internal fixation in humeral diaphyseal fractures for 2800 matched patient pairs utilizing the Nationwide Readmissions Database
Source: J Orthop Surg Res. 2023 Jun 20;18:442. doi: 10.1186/s13018-023-03663-2 (PMC10280891; doi:10.1186/s13018-023-03663-2)
Supplement: Supplementary file 1 — Additional file 1. Table 1. ICD-10 Codes for Identifying IMN and ORIF Cases. [file 13018_2023_3663_MOESM1_ESM.docx]

**Supplemental Table 1.** ICD-10 Codes for Identifying IMN and ORIF Cases

|  | **Insertion** | | | **Reposition^*^** | | |
| --- | --- | --- | --- | --- | --- | --- |
|  | **Open Approach** | **Percutaneous Approach** | **Percutaneous Endoscopic Approach** | **Open Approach** | **Percutaneous Approach** | **Percutaneous Endoscopic Approach** |
| **Intramedullary Nail Fixation** |  |  |  |  |  |  |
| Right | 0PHF06Z | 0PHF36Z | 0PHF46Z | 0PSF06Z | 0PSF36Z | 0PSF46Z |
| Left | 0PHG06Z | 0PHG36Z | 0PHG46Z | 0PSG06Z | 0PSG36Z | 0PSG46Z |
| **Open Reduction Internal Fixation** |  |  |  |  |  |  |
| Right | 0PHF04Z | 0PHF34Z | 0PHF44Z | 0PSF04Z | 0PSF34Z | 0PSF44Z |
| Left | 0PHG04Z | 0PHG34Z | 0PHG44Z | 0PSG04Z | 0PSG34Z | 0PSG44Z |

*****Refers to moving the humeral diaphyseal fractured bone to a new, or more advantageous, positioning - does not refer to a revision
